# Therapeutic Approaches for the Prevention of Upper Limb Repetitive Strain Injuries in Work-Related Computer Use: A Scoping Review

**DOI:** 10.1007/s10926-024-10204-z

**Published:** 2024-06-06

**Authors:** Alita de Waal, Amy Killian, Afika Gagela, Jada Baartzes, Susan de Klerk

**Affiliations:** Division of Occupational Therapy, Department of Health and Rehabilitation Science, Faculty of Medicine and Health Science, Stellenbosch University, Cape Town, 8000 South Africa

**Keywords:** Therapeutic uses, Primary prevention, Upper extremity, Cumulative trauma disorders, User-computer interface

## Abstract

**Purpose:**

To explore and describe therapeutic approaches for the prevention of upper limb (UL) repetitive strain injuries (RSI) amongst computer users in the twenty-first century.

**Methods:**

A scoping review was conducted using the method described by Arksey and O’Malley, further enhanced by Levac et al. to ensure rigor, validity and reliability during analysis. Key concepts pertaining to the research question have been mapped, following comprehensive searches of relevant electronic databases namely EBSCOHost (Academic Search Premier, CINAHL, eBook Collection, E-Journals, Health Source-Consumer Edition, Health Sources—Nursing/Academic Edition and MEDLINE), PUBMED and Google Scholar. The identified studies have been presented in a descriptive numerical summary to address the research aim.

**Results:**

From the 577 studies initially identified, 58 studies were eligible for inclusion in the scoping review after abstract and full text screening. Strategies for the prevention of UL RSIs in computer users were categorised into overarching types of intervention as well as the factors which contribute towards sustained implementation of prevention strategies. Using ergonomic equipment was the most prevalent approach during intervention, breaks and rest periods were found to be the less common intervention offered to prevent RSIs. The majority of the studies noted personal worksite adjustments, including adjustments of the chair, back rest, lumbar support, handles or any arm support to the individual as a strategy to prevent UL RSIs. In high income countries the use of ergonomic equipment was the most common type of approach during intervention, in middle income countries stretches were the most common therapeutic intervention strategy and in low-income countries there was an even distribution between a number of different therapeutic interventions aimed at preventing RSIs.

**Conclusions:**

The review provides an overview of approaches and a comprehensive baseline for identifying further research required to generate prevention approaches. The information within the review may be used to impact company practice, policy and decision making in terms of developing prevention strategies.

## Introduction

The rapid development of a digitally driven world in the twenty-first century has brought with it an increase in the use of computers in all spheres of life [1]; the introduction of computers has played a significant role in the organisation of work within the information sector of economies. The increase in the number of employees working with a computer and mouse has led to a consequent increase in the number of work-related repetitive strain injuries (RSIs) [2]. This is presumed to be as a result of individuals working for lengthy periods of time using a personal computer (PC), which requires the maintenance of a static posture of the upper body to ensure adequate positioning of the neck and upper limbs (UL) [2]. The repetitive movements required for typing, associated jerky movements and excessive force when typing on a keyboard are contributing factors to UL pain and RSI [3]. General symptoms of RSI, additionally referred to as cumulative trauma injuries, include tenderness in the affected muscles which can result in a loss of strength, throbbing or a tingling sensation in the affected area, and possible localised loss of sensation [4].

The high cost of healthcare, work absenteeism, decrease in productivity, and employee turnover has created a demand for implementing preventative and rehabilitative interventions in the workplace [5].

This scoping review maps the available information on therapeutic approaches for the prevention of UL RSIs in computer users. In the context of the current review, therapeutic approaches are operationalised to include therapeutic programs, intervention strategies and therapeutic strategies. This information can be used as a reference point for therapists (including but not limited to occupational and physiotherapists), employers and computer users. It adds to the body of knowledge for further research into the frequent occurrence of this health condition secondary to work-related computer use. Study findings may inform policy makers, employers and employees secondary to the increased number of people working from home post the COVID-19 pandemic [6]. The scoping review therefore aimed to explore and describe the research conducted in the twenty-first century (2000–2021) on therapeutic approaches for the prevention of UL RSIs amongst computer users. The objective of the study was to (1) identify and describe therapeutic approaches employed to prevent UL RSIs in computer users as per literacy, (2) identify factors which contribute towards the sustained implementation of therapeutic approaches of UL RSI prevention strategies in computer users and (3) identify and describe potential trends and gaps in literature relating to therapeutic prevention approaches for UL RSIs in computer users.

## Methods

The study adhered to scoping review methodology for the rigorous collection, synthesis, appraisal and presentation of the findings from existing research on the topic under investigation [7]. Scoping reviews are referred to as ‘a mapping process’ since they summarise a range of evidence in order to convey the breadth and depth of a field. A scoping review can include a range of study designs and address complex and diverse questions that cannot typically be addressed with a systematic review.

The stages of the scoping review were:

1. Identifying the Research Question

Scoping review methodology allows a post hoc narrowing of the research question and adoption of the criteria set a priori. The research question was formulated considering the PCC (Population, Concept, Context) mnemonic and as a result the Population were identified as computer users with UL RSIs, the Concept is therapeutic strategies to prevent UL RSIs and the Context the workplace environment in the twenty-first century [7]. The questions were:

- *What are the therapeutic approaches used to prevent RSIs in the UL in computer users within the workplace environment in the twenty-first century*?
- *What factors contribute towards the implementation of therapeutic approaches aimed at preventing RSIs of ULs amongst computer users within the workplace*?
- *What are the trends and gaps in literature of in twenty-first century relating to therapeutic approaches to aid prevention of RSI of the UL in computer users*?

2. Identifying Relevant Studies

Ensuring relevance of included studies, electronic databases were identified with the assistance of an expert librarian. These included EBSCOHost (Academic Search Premier, CINAHL, eBook Collection, E-Journals, Health Source-Consumer Edition, Health Sources—Nursing/Academic Edition and MEDLINE), PUBMED and Google scholar. Published and Grey literature from the year 2000 to 2022 was considered.

An initial search string was constructed with assistance of the expert librarian. Search terms were selected in alignment with the research objectives and questions to obtain literature which would provide insight into therapeutic approaches used to prevent UL RSIs in computer users. The breadth of studies included was further refined in accordance with the inclusion and exclusion criteria, to confirm eligibility for the inclusion to the review.

Medical subject headings (MeSH) were used to identify search terms, allowing for a comprehensive literature search (see Table 1). Boolean operators’ conjunction functions ‘AND’ and ‘OR’ were used to narrow the search. In addition, it was stipulated where the search term should be included in headings and introduction found within the studies to allow for a comprehensive search. Following consideration against eligibility criteria and subsequent inclusion, a manual search commenced. Researchers worked through reference lists of included documents to filter out literature to consider against the eligibility criteria. This process was concluded when saturation of literature was reached with no new literature found when performing advance searches.

3. Selecting Studies for Analysis (384 words)

**Table 1:** Search terms used (including Boolean operator)

Title/Abstract	“Prevention” OR “Intervention” OR “Treatment” OR “Programme” OR “Therapeutic Strategy”
AND Title/Abstract	“Upper limbs” OR “Upper extremities” OR “Hand” OR “Arm” OR “Forearm” OR “Upper Arm”
AND Title/Abstract	“Repetitive Strain Injury” OR “Occupational Overuse Injury” OR “Cumulative Trauma Disorder” OR “Strain Injury, repetitive” OR “Motion disorder, repetitive”
AND Title/Abstract	“Computer” OR “Computer users” OR “Computer work” OR “Computer related”

The identified studies were imported into Covidence online software designed to assist researchers in conducting scoping and systematic reviews. Firstly, duplicates were removed, after which title and abstract screening commenced. Two members of the research team screened titles and abstracts against eligibility criteria, to limit bias and individual errors. These inclusion and exclusion criteria can be found in Table 2. Some of the inclusion criteria categories included the written language of the study, the type of study, from which database the study was retrieved and the health outcome of the study. Studies were excluded based on the reason for computer use at home and information published on blogs, webpages and seminars or conferences.

**Table 2:** Eligibility criteria

	Inclusion	Exclusion
Language	Studies published in English	
Type of study	Full text studies and grey literature which was available on the stipulated online databases Published and unpublished quantitative and qualitative studies Worldwide literature including data from low, lower-middle, upper-middle- and high-income countries Systematic reviews; meta-analyses of randomised control trials; controlled trials, cohort studies; case series; individual case studies	Blogs; seminars; webpages; conferences
HEALTH OUTCOME	Studies which included musculoskeletal disorders (WMSD) affecting the human shoulder, arm, forearm, wrist and hand associated with computer work exposures	Studies conducted involving computer users who do not use the computer to complete work-related tasks, i.e. people making use of a computer for leisure activities Content relating to other fields of occupational therapy, physical therapy or hand therapy which do not relate to the upper limb Therapeutic prevention strategies preventing injuries which are not as a result of RSI
Intervention	Interventions to prevent upper extremity WMSD associated with computer use Prevention strategies proposed by occupational therapists, physiotherapists and/or hand therapists	
Study population	Computer or VDT users for work	
Moderating factors	Studies which describe factors that facilitate implementation of preventive therapeutic strategies	

**Fig. 1:**
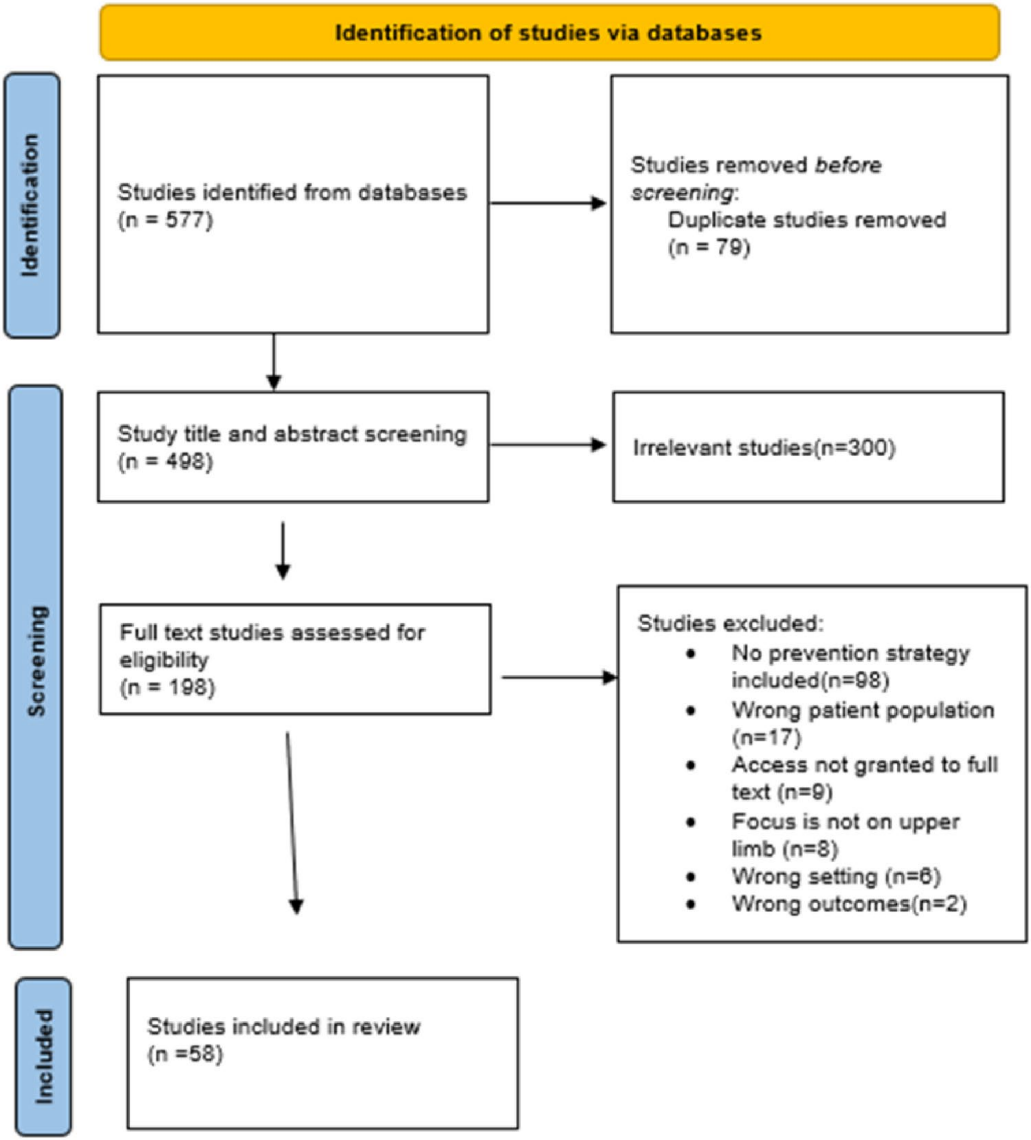
PRISMA flow diagram

Conflicts between the reviewers were resolved by a third, independent research team member towards a final decision. The PRISMA flow diagram (Fig. 1) depicts the literature search results.

4. Charting the Data

A draft data charting table was compiled and piloted by the research team with three randomly selected studies. During the process of piloting, edits were made and finalised on the draft data charting table. After the research team confirmed that the draft data charting table was extensive for data collection the data was charted and checked for accuracy by all members of the research team. All included studies were captured using the data charting table. This process assisted to categorise included documents into specific themes and ensured consistency of data extraction amongst researchers, both of which are crucial factors in presenting a comprehensive and valid scoping review [8].

5. Collating, Summarising and Reporting the Results

A descriptive numerical summary of the included studies is reported in the results section of this review. To provide a comprehensive overview of currently available research, tables, graphs and figures were constructed to display the distribution of countries in which research has been conducted and the most prevalent therapeutic prevention approaches and potential factors which may act as barriers to the implementation of these strategies.

Finally, results are explained in terms of how they relate to specific concepts as it pertains to preventing UL RSIs in computer users, addressing the review questions. Implications for research include recommendations for future research in terms of the research gaps identified.

## Results

Five hundred and seventy-seven studies were initially identified from databases and imported into Covidence after which 79 duplicates were removed. After the title and abstract screening, 300 studies were removed and as a result 198 studies were eligible for full text screening. During full text screening documents were excluded for the following reasons: 98 documents did not include a prevention strategy, 17 considered a different patient population, 8 studies did not focus on the UL, in 6 studies the setting did not match inclusion criteria, in 2 studies outcomes did not match inclusion criteria and in 9 instances full text access was not granted.

As a result, 58 studies were included in the review and included 15 randomised control trials [9–23], 11 non-randomised experimental studies [24–34], 7 scoping reviews [4, 35–40], 11 systematic reviews [3, 41–50] and 14 studies comprising of a variety of designs (including cohort study, prevalence study, cross sectional study, case report, qualitative research report and case controls) [51–64] (see data extraction in Table 3).

**Table 3:** Extracted Data

Author Year	Title	Country (income status)^a^	Study design (*n*)	Type of therapeutic intervention	Specific therapeutic intervention	Aim of the study	Results/conclusions
Alhay, B 2018	An analysis of the kinematics of the elbow and wrist joints, and the muscle activity of the arm when using three different computer mice [41]	United Kingdom (high income)	Systematic review	Ergonomic equipment	Mouse design	The aim of this study was to establish how the design of a computer mouse influences the posture, biomechanics and muscle activity of the forearm	In conclusion, the design of the mouse plays an important role in the position of the forearm, posture deviation in the forearm and whole body, wrist ulnar deviation and an average and/or maximum usage of wrist extensors, as it could increase the risk of musculoskeletal disorders
Goodman, G; Kovack, L; Fisher, A; Elsesser, E; Bobinski, D; Hansen, J 2012	Effective interventions for cumulative trauma disorders of the upper extremity in computer users: practice models based on systematic review [3]	USA (high income)	Systematic review	– Ergonomic equipment – Preventative ergonomic training – Break and rest periods	Forearm support, alternative keyboard; adapted computer mouse	Determining the effectiveness of current interventions that focus on cumulative trauma disorders (CTD)-related symptoms of the upper extremities in computer users and how these methods are put into practice	In conclusion, there isn’t one intervention technique that would successfully decrease CTD symptoms and upper extremity discomfort of workers using the computer. There are two models that were created to assist members of the multiple disciplinary teams to a variation of many intervention types for computer users suffering with CTD
Hoe, V CW; Urquhart, DM; Kelsall, HL; Sim, MR 2013	Ergonomic design and training for preventing work-related musculoskeletal disorders of the upper limb and neck in adults [42]	USA (high income)	Systematic review	– Ergonomic equipment – Ergonomic work environment – Preventative ergonomic training	Ergonomic keyboard and mouse, arm support	To assess the effects of workplace ergonomic design or training interventions, or both, for the prevention of work-related upper limb and neck MSDs in adults	In conclusion, evidence was found that when using arm support with an alternative mouse, it can decrease the chances of getting neck/shoulder musculoskeletal disorders. There is moderate evidence found stating that neck/shoulder and right upper limb musculoskeletal disorders are decreased when differentiating an alternative or conventional mouse with and without arm support
Verhagena, AP; Karelsa, C; Bierma-Zeinstraa, SMA; Feleusa, A; Dahaghina, S; Burdorfb, A; Koes, BW 2007	Exercise proves effective in a systematic review of work-related complaints of the arm, neck, or shoulder [43]	Netherlands (high income)	Systematic review	– Ergonomic equipment – Preventative ergonomic training – Stretches – Breaks and rest periods	Adapted keyboards	To summarise the existing knowledge and evidence concerning the efficacy of frequently performed interventions in work-related upper extremity musculoskeletal disorders (CANS)	In conclusion, there is not much evidence for the efficacy of certain keyboards with different force displacement or geometry only for patients with carpal tunnel syndrome. There is also not much evidence that shows the benefit of exercises when compared to massages, adding breaks during the time spent working on computers, massage as additional treatment to manual therapy and manual therapy as additional treatment to exercises in patients with non-specific work-related complaints
Lincoln, A; Vernick JS; Ogaitis, S; Smith, GS; Mitchell, CS; Agnew, J 2000	Interventions for the primary prevention of work-related carpal tunnel syndrome [44]	USA (high income)	Systematic review	Ergonomic equipment	Alternative keyboards, computer mouse designs, wrist supports, keyboard support systems, workstation re-design	To evaluate interventions for the primary prevention of work-related carpal tunnel syndrome	In conclusion, studies agree that implementing multiple components into ergonomic programs, different key supports, and mouse and tool redesign may be valuable in the reduction of RSI
Kirk, E; Strong, J 2008	Management of eWork health issues: a new perspective on an old problem [45]	Australia (high income)	Systematic review	Ergonomic equipment	N/A	The aim of the study is to present a brief overview of acknowledged risk factors and their associated health concerns and predicted costs, both to individuals and to industry	In conclusion, computer users experienced an extensive amount of discomfort and injury. Contact centres make a perfect partner to develop and trial innovative programs to decrease WRULDs and CVSs related to economic load
Andersen, JH; Fallentin, N; Thomsen, JF; Mikkelsen, S 2011	Risk factors for neck and upper extremity disorders among computers users and the effect of interventions: an overview of systematic reviews [46]	Germany (high income)	Systematic review	– Ergonomic work environment – Preventative ergonomic training	N/A	To provide a synthesis of the evidence on computer work and the risk of carpal tunnel syndrome and UEMSDs and the effect of workplace interventions	In conclusion, there was average to above average evidence that shows an increased risk of acute or transient pain complaints amongst the computer users, when using the mouse intensively. There were no results of preventive interventions that include only workstations. There was very restricted evidence that a combination of ergonomics training and workplace adjustments could be valuable
Gasibat, Q; Simbak, NB; Aziz, AA 2017	Stretching exercises to prevent work-related musculoskeletal disorders: a review article [47]	Malaysia (upper middle income)	Systematic review	Stretches	Stretches for the forearm muscles	The aim of this study was to synthesise the recent literature on workplace stretching exercise programs and their effects on reducing work-related MSDs in different occupational groups	In conclusion, this study has proven that there isn’t enough evidence to prove that stretching at work would help prevent work-related musculoskeletal injuries, but rather that doing stretches can assist in decreasing discomfort/pain and increasing range of motion
Leonard-Dolack; Goodman, G; Kovach, L; Fisher, A; Elsesser, E; Bobinski, D; Hansen, J 2010	The effectiveness of intervention strategies used to educate clients about prevention of upper extremity cumulative trauma disorders [48]	USA (high income)	Systematic review	– Ergonomic equipment – Ergonomic work environment	Policy of work environment	This paper examines the effectiveness of different intervention strategies used to educate workers about the prevention of upper extremity CT	Intervention strategies may include discussion, demonstration, or practice of proper posture and body mechanics. Practice of correct techniques may not be necessary to achieve improved hand-use patterns in assembly-line workers. However, practice with intensive feedback may be necessary to achieve the best performance of hand-wrist position, but not sitting posture, in computer operators. The effects of practice on correct hand use may depend on the type of job task. Further research is needed to clarify the role of practice and feedback in CTD prevention
Brewer, S; Van Eerd, D; Amick III, BC; Irvin, E; Daum, KM; Gerr, F; Moore, JS; Cullen, K; Rempel, D 2006	Workplace interventions to prevent musculoskeletal and visual symptoms and disorders among computer users: a systematic review [49]	USA (high income)	Systematic review	– Ergonomic equipment – Ergonomic work environment – Preventative ergonomic training – Breaks and rest periods	Alternative pointing device (track ball and mouse), alternative keyboard, arm supports	To identify published studies that evaluated the effects of workplace interventions on visual or upper body musculoskeletal symptoms or disorders among computer users	In conclusion, there is an average amount of evidence that adjusting workstations has no effect on musculoskeletal disorders. There was average evidence about employees taking rest breaks and exercising through the break, but still no effect on the musculoskeletal outcomes. Evidence shows that alternative pointing devices have a good effect on musculoskeletal outcomes. There were mixed findings about whether ergonomic training, arm supports, alternative keyboards and rest breaks influence musculoskeletal outcomes
Van Eerd, D; Munhall, C; Irvin, E; Rempel, D; Brewer, S; van der Beek, AJ; Dennerlein, JT; Tullar, J; Skivington, K; Pinion, C; Amick, B 2015	Effectiveness of workplace interventions in the prevention of upper extremity musculoskeletal disorders and symptoms: an update of the evidence [50]	Canada (high income)	Systematic review	– Ergonomic equipment – Ergonomic work environment – Preventative ergonomic training – Promotive education – Stretches – Breaks and rest periods	Wrist support, vibrating feedback on mouse, workplace resistance training	To measure the level of evidence to support intervention strategies for RSI	No intervention evaluations produced negative effects (e.g., increased symptoms or lost time claims). Stretching exercise programmes, vibration feedback on mouse use and workstation forearm supports had a moderate level of evidence for a positive effect in preventing UEMSD. Practitioners should consider *implementing stretching exercise programmes, vibration feedback on mouse use or workstation forearm supports in practices if applicable to the work context* Resistance training programmes had a strong level of evidence. We recommend *implementing a workplace-based resistance training exercise programme to help prevent and manage UEMSD symptoms and disorders*
Bruno Garzaa, L; Youngb, JG 2014	A literature review of the effects of computer input device design on biomechanical loading and musculoskeletal outcomes during computer work [35]	USA (high income)	Scoping review	Ergonomic equipment	Alternative keyboards and mouse	The objective of this paper was to review studies specifically describing the biomechanical loading and/or musculoskeletal outcomes associated with conventional and alternative input devices for use in a typical office desktop scenario	In conclusion, not all computer pointing devices or keyboards produced equivalent biomechanical responses in users. Some alternative pointing devices and keyboards were associated with decreased hand and shoulder biomechanical loading. These alternative input devices may be effective at preventing or reducing the severity of musculoskeletal outcomes among computer users
Staal, JB; de Bie, RA; Hendriks, EJM 2007	Aetiology and management of work-related upper extremity disorders [36]	Netherlands (high income)	Scoping review	– Ergonomic equipment – Preventative ergonomic training – Promotive education	Forearm support, ergonomic training, exercise, alternative keyboard	– Reviews the clinical manifestations, mechanisms and aetiology of work-related upper extremity disorders through an exploration of the literature Also examines and discusses the evidence for the effectiveness of several preventative and therapeutic interventions	The studies found that the use of forearm support in combination with ergonomics training was protective for the occurrence of neck, shoulder and upper extremity pain and it resulted in fewer reported symptoms in the neck and back The benefits of exercise in the prevention of Work-Related Upper Extremity Disorder (WRUED) have not been clearly supported yet by RCTs although it has been found to be protective for upper limb symptoms Limited evidence for the effectiveness of exercises and for the use of keyboards with an alternative force–displacement of the keys or an alternative geometry
Szabo, R; King, K 2000	Current concepts review. Repetitive stress injury: diagnosis or self-fulfilling prophecy [37]	USA (high income)	Scoping review	– Ergonomic equipment – Stretches – Preventative ergonomic training	Stretching, alternating tools and exercises	To assess whether repetitive stress injuries are formally diagnosed or individual self-fulfilling prophecy and to review the current concepts of RSI	With musculoskeletal injuries, one frequently prescribes a rehabilitative program that includes stretching and muscle-strengthening, alteration of tools, and aerobic conditioning
Maruthappapandian, J; Gnana Chellaiyan, V; Ali, FL; Avinash, D 2019	Healthy workplace with ergonomics among software engineers: a review [38]	India (lower middle income)	Scoping review	– Ergonomic equipment – Ergonomic work environment – Preventative ergonomic training – Breaks and rest periods	Rest breaks, comfortable positioning, adjustable chair (with back support), adapted keyboard	The objective of this review is to discuss the role or principles of ergonomics on preventing musculoskeletal problems among software engineers	Many studies showed that an improvement in ergonomic practice and regular rest in between work can avert musculoskeletal problems Maintaining a comfortable posture and standing or extending in between the work is essential in preventing muscle fatigue The chair should have an adjustable height with back support (both upper and lower back) The keyboard platform should be such that when the fingers are positioned on the home row keys, their upper border should be the same height with the elbow or slightly lower, but not higher
Goyal, K; Balodhi, A; Manglik, P; Mohd Asif, D; Rai, RH; Fahim, T 2020	Minimising the adverse effects of work environment in upper limb: a literature review [39]	India (lower middle income)	Scoping review	– Ergonomic equipment – Ergonomic work environment – Preventative ergonomic training – Promotive education – Stretches – Breaks and rest periods	– Ergonomic equipment: (keyboard; arm rests) Promotive education: (Neck, shoulder, upper back seated exercises) (20–20 rule) – Ergonomic setting up of workstation (neutral sitting posture and proper alignment; height of workstation table) – Rest breaks, stretches, and exercises	The study’s aim is to focus on improving quality of life through various intervention strategies within the work organisation thus enhancing work quality and output	In this study, it was concluded that education about correct rest intervals, seated stretching exercises, proper posture to avoid injury, ergonomic changes and isometric exercises are the most beneficial interventions to prevent RSI in the UL
Kaur Karir, H 2020	Role of ergonomics in inducing dynamicity by transforming sedentary computer workstation [40]	Poland (high income)	Scoping review	Ergonomic equipment	Interchanging mouse designs	The study aims to analyse the prolonged sedentary position from both a biological and ergonomic perspective	This research found that interchanging mouse designs while using the computer throughout the day could be the key to reducing the risk of RSI
Taylor, K 2002	Research on RSI and Breaks [4]	New Zealand (high income)	Scoping review	– Promotive education – Breaks and rest periods	Breaks and micropauses	To provide background information and research on Work-Related Musculoskeletal Disorders (WMSD) and focuses on the scientific evidence supporting introduced breaks for computer users to prevent and manage musculoskeletal disorders	This paper shows evidence that common computer-related MSDs can be prevented and remedied by adopting a regular regime of breaks and micropauses. This has the effect of reducing the user’s muscle fatigue which contributes to the initial development of symptoms, and cancels the associated reduction in performance caused by fatigue. As a preventative measure, this protects the user from sustaining long-term injury at a reduced cost to the employer
Rempel, DM; Krause, N; Goldberg, R; Benner, D; Hudes, M; Goldner, GU 2005	A randomised controlled trial evaluating the effects of two workstation interventions on upper body pain and incident musculoskeletal disorders among computer operators [9]	USA (high income)	RCT (*n* = 128)	– Ergonomic equipment – Promotive education	Forearm support board and a trackball	The aim of this study was to determine whether two simple workstation interventions—forearm support board or a trackball—when used by computer-based customer service workers, would reduce the incidence of upper body musculoskeletal disorders and pain severity. Secondary aims included estimating the effects of the intervention on productivity and costs	Subjects in the intervention groups reported decreased pain in comparison to the control group. Hence, providing a large forearm support combined with ergonomic training, is an effective intervention to prevent upper body musculoskeletal disorders and reduce upper body pain associated with computer work among call centre employees. No significant differences between the two groups in terms of productivity was reported. Mixed effects of using trackball reported
Conlon, CF; Krause, N; Rempel, DM 2009	A randomised controlled trial evaluating an alternative mouse or forearm support on change in median and ulnar nerve motor latency at the wrist [10]	USA (high income)	RCT (*n* = 154)	Ergonomic equipment	Forearm board and alternative mouse	The purpose of this study was to determine the effects of an alternative mouse and/or a forearm support board on nerve function at the wrist among engineers	Forearm board use had no protective effect for the median nerve while the use of an alternative mouse had a protective effect on ulnar nerve function at the wrist
Zecevic, A; Miller, DI; Harbur, K 2010	An evaluation of the ergonomics of three computer keyboards [24]		NRCT (*n* = 16)	Ergonomic equipment	– ADAPTED KEYBOARDS – The fixed alternative keyboard featured a split angle of 12° and a moderate lateral inclination angle of 10° – The adjustable OPEN alternative keyboard was used with a 15° split setting, which resulted in a marked 42° of demiboard lateral inclination	To explore any significant differences between ergonomic keyboards (open and fixed) and a standard keyboard for computer users	The standard keyboard is more likely to cause musculoskeletal injury as it places the hand in an awkward position for longer periods. The fixed alternative keyboard enabled natural hand position and thus has a proven potential to improve hand posture and thereby reduce the risk of developing cumulative trauma disorders of the wrist due to keyboard use
Delisle, A; Larivière, C; Plamondon, A; Imbeau, D 2006	Comparison of three computer office workstations offering forearm support: impact on upper limb posture and muscle activation [25]	Canada (high income)	NRCT (*n* = 18)	– Ergonomic work environment – Breaks and rest periods	Adapted workstation, adjustable arm rests or adjustable chair	The aim of this study was to investigate the effect of different workstations, all offering the possibility of forearm support, on upper limb muscle activation and posture while working alternately with the keyboard and computer mouse	Leaning the forearms alternately on the work surface and on the chair-armrests (on a daily or weekly basis), using an easily adjustable workstation, can be seen as a way of alternating the muscles solicited during computer work. Such variation in work posture proved to be promising for preventing musculoskeletal disorders in computer work
McLean, L; Tingley, M; Scott, RN; Rickards, J 2000	Computer terminal work and the benefit of microbreaks [11]	Canada (high income)	RCT (*n* = 15)	Breaks and rest periods	‘Ergobreak’ computer program to remind workers to take microbreaks	– To investigate myoelectric signal (MES) activity and perceived discomfort in areas of common CTD complaints: the neck, the low back, the shoulder region, and the wrist – To determine the effect of microbreak protocols on muscle activation behaviour – To determine the effect of microbreaks on perceived discomfort – To determine the effect of microbreaks on worker productivity	It was more beneficial to take microbreaks according to a fixed schedule (every 20 min for the back, shoulder, and forearm, and every 40 min for the neck) than it is to simply take breaks when one felt a break was necessary as this resulted in a slow development of discomfort in the neck, low back, shoulder, and wrist areas
Simoneau, GG; Marklin, RW; Berman, JE 2003	Effect of computer keyboard slope on wrist position and forearm electromyography of typists without musculoskeletal disorders [27]	USA (high income)	NRCT (*n* = 16)	Ergonomic equipment	QWERTY keyboard with slopes at positive and negative angles	To determine the effect of computer keyboard slope angle on forearm musculature EMG activity in individuals without any upper-extremity symptoms of MSDs	Wrist extension decreased as the keyboard slope decreased. Furthermore, a slight decrease in percentage of maximum voluntary contraction (MVC) of the extensor carpi ulnaris (ECU) muscle was noted as the keyboard slope decreased. This data suggests that a keyboard with a neutral (horizontal) slope or a keyboard with a downward slope might have beneficial effects to prevent or treat upper-extremity injuries related to the frequent use of computer keyboards
Andersen, CH 2012	Effect of different training regimes on musculoskeletal pain in neck and shoulder [12]	Denmark (high income)	RCT (*n* not reported)	Preventative ergonomic training	Strength exercise training	The aim of this PhD project was, in an exercise evaluation study and two intervention studies, to investigate effects of contrasting types of intensive muscle training on pain, disability and strength in office workers with nonspecific neck and shoulder pain	Traditional strength training exercises for the neck and shoulder as well as exercises commonly recommended by physical therapists effectively relieve neck and shoulder pain. Results emphasise that both fewer and longer as well as more and shorter sessions of high-intensity training provides pain relief. Importantly, the result of the present thesis provides flexibility for companies and employees regarding individual preferences for exercise selection and time-wise distribution when implementing specific training exercises in an effective manner into a weekly work schedule
Joshi, V; Bellad, A 2011	Effect of yogic exercises on symptoms of musculoskeletal disorders of upper limbs among computer users: a randomised controlled trial [17]	India (lower middle income)	RCT (*n* = 58)	– Promotive education – Stretches	Yoga exercises	Evaluate effectiveness of yogic exercises in the improvement of symptoms of MSDs of upper limbs	There was significant reduction in symptom severity score and improvement in functional status score in yoga with counselling group when compared to only counselling group. There is also a significant decrease in self-reported symptoms like CT myalgia symptom and improvement in weakness
Meijer, EM; Sluiter, JK; Frings-Dresen, MHW 2008	Effectiveness of a feedback signal in a computer mouse on upper extremity musculoskeletal symptoms: a randomised controlled trial with an 8-month follow-up [16]	Netherlands (high income)	RCT (*n* = 354)	Ergonomic equipment	Computer mouse with feedback signal	To study the effectiveness of using a computer mouse with a feedback signal for upper extremity musculoskeletal symptoms in office workers	Results show that the prevalence and incidence of upper extremity musculoskeletal symptoms did not differ between the intervention group and the control group at 4 and 8 months after baseline. The intervention group did report less disability compared with the control group in both the total group and the subgroup of office workers who reported upper extremity musculoskeletal symptoms at baseline. Hence the use of the feedback signal computer mouse does not affect the prevalence and incidence of upper extremity musculoskeletal symptoms, but it does lower disability scores
Spekle, EM; Hoozemans, MJM; Blatter, BM; Heinrich, J; van der Beek, AJ; Knol, D; Bongers, PM; van Dieen, JH 2010	Effectiveness of a questionnaire-based intervention programme on the prevalence of arm, shoulder and neck symptoms, risk factors and sick leave in computer workers: a cluster randomised controlled trial in an occupational setting [15]	Netherlands (high income)	RCT (*n* = 741)	– Ergonomic work environment – Promotive education	Workplace furniture	To assess the effectiveness of this intervention programme on the prevalence of arm, shoulder and neck symptoms, reduction of exposure to risk factors, and sick leave in a population of computer workers	Significant positive effects as to an increase in receiving education and a decrease in exposure to adverse postures and movements. No significant effects found for most risk factors of arm, neck, shoulder related to sick leave taken
Thennarasi, M. 2015	Effectiveness of ergonomics on physical discomfort among computer users at selected it office in Madurai [28]	India (lower middle income)	NRCT (*n* = 40)	– Ergonomic work environment – Preventative ergonomic training	Not specified	1. To assess the level of physical discomfort among computer users in a selected IT office 2. To evaluate the effectiveness of ergonomic interventions among computer users in selected IT office 3. To determine the association between the levels of physical discomfort with selected socio demographic variables	The demonstration of ergonomic intervention was effective in reducing the levels of physical discomfort among computer users No significant association between socio-economic demographic variables and levels of physical discomfort among computer users in this study
van Galen, GP; de Haan Ab, LH 2007	Effects of a vertical keyboard design on typing performance, user comfort and muscle tension [26]	Netherlands (high income)	NRCT (*n* = 9)	Ergonomic equipment	Yogitype keyboard	To investigate the Yogitype concept as to the user’s typing performance, comfort and health	There’s no significant difference in task performance between the Yogitype keyboard and the standard keyboard. Yogitype keyboard posture was rated as being more comfortable than the posture for the standard keyboard. Overall muscle activation was higher when working with the traditional keyboard
Ramalingam, KP; Karthikeyan, P; van Lieshout, J; Akiro, C; Wohemani, R; Girey, M 2010	Effects of exercise intervention on work-related musculoskeletal discomforts among computer users [29]	Papua New Guinea	NRCT (*n* = 39)	– Promotive education – Preventive ergonomic training – Stretches – Breaks and rest periods	Exercise, breaks and posture education	The study aimed to identify work-related musculoskeletal disorders discomforts from using computers and to explore the effect of exercise to decrease such discomforts	The study suggests that long-term use of computers is associated with various musculoskeletal discomforts and exercise, rest breaks and posture correction seem to alleviate or reduce the discomforts
van den Heuvel, SG; de Looze, MP; Hildebrandt, VH; The, KH 2003	Effects of software programs stimulating regular breaks and exercises on work-related neck and upper-limb disorders [23]	Netherlands (high income)	RCT (*n* = 219)	– Stretches – Breaks and rest periods – Preventative ergonomic training	Exercise and breaks	This study evaluated the effects on work-related neck and upper-limb disorders among computer workers stimulated (by a software program) to take regular breaks and perform physical exercises. Possible effects on sick leave and productivity were studied as well	Pre- versus post-intervention scores of severities and frequency did not reveal any differences between the control and intervention groups, whereas the results concerning (post-intervention) perceived recovery and revealed a favourable effect for the stimulation of regular breaks There seems to be no additional effects from performing physical exercises during these breaks
Voerman, GE; Sandsjo, L; Vollenbroek-Hutten, MMR; Larsman, P; Kadefors, R; Hermens, HJ 2007	Evaluating the effectiveness of educational training program about repetitive strain injury on computer user employees at Damanhour University [13]	Sweden and Netherlands (high income)	RCT (*n* = 79)	Promotive education	Myofeedback training and ergonomic counselling	To investigate the effects of ambulant myofeedback training including ergonomic counselling (Mfb) and ergonomic counselling alone (EC), on work-related neck-shoulder pain and disability	4 weeks of intervention significantly reduced pain intensity and disability, and this effect remained after 3- and 6-month follow-up. Myofeedback training combined with ergonomic counselling is thus beneficial for female computer workers over the age of 45, reporting pain and disability in the neck-shoulder region. No differences were observed between the Mfb and EC group for outcome and subjects in both intervention groups showed comparable chances for improvement in pain intensity and disability
Peper, E; Gibney, KH, Wilson, VE 2004	Group training with healthy computing practices to prevent repetitive strain injury (RSI): a preliminary study [30]	USA (high income)	NRCT (*n* = 28)	– Preventative ergonomic training – Promotive education – Breaks and rest periods	– Micro-break: dropping one’s hands on the lap and reducing forearm muscle tension for 1 or 2 s – Meso-break: stopping to stretch or do total body movement for 5–20 s – Macro-break example: taking time out to go for a walk for a few minutes – Break reminder program – Ergonomic keyboards, chairs and keyboard trays – Diaphragmatic breathing	This pilot study investigated whether group training, in which participants become role models and coaches, would reduce discomfort as compared to a nontreatment Control Group to determine if healthy computing concepts taught in a group setting would reduce symptoms and improve work style	After 6 weeks, the Experimental Group as compared to the Control Group reported a significant overall reduction in work-related symptoms – The use of individualised sEMGs at the workstation allowed the participants to see and hear their covert muscle tension. They appeared to use this to encourage mastery of neck and shoulder relaxation, slower breathing, and taking micro-breaks while working at the computer
Callegari, B; Maniglia, M; da Silva Filho, M 2017	Hand rest and wrist support are effective in preventing fatigue during prolonged typing [31]	Brazil (upper middle income)	NRCT (*n* = 25)	Ergonomic equipment	Hand rest and wrist support	Aimed to investigate whether the duration of typing and the use of two strategies (hand rest and wrist support) changes muscle physiological response and therefore the electromyography records	Hand rest and wrist support can successfully reduce muscle fatigue in specific upper limb muscles during prolonged typing, leading to a muscle-selective reduction in the occurrence of fatigue and thus provide direct evidence that they may prevent work-related musculoskeletal disorders
Hiremath, PKS 2015	A study to assess the effect of self-instructional module on knowledge regarding prevention of occupational health hazards in Pune City [32]	India (lower middle income)	NRCT (*n* = 50)	– Ergonomic equipment – Ergonomic work environment – Promotive education – Stretches	Chair arrangements, office lighting, education and correction on knowledge	1. To assess the existing knowledge on occupational health hazards among computer operators 2. To evaluate the effect of a self-instructional module on knowledge of occupational health hazards among computer operators 3. To correlate the knowledge on occupational health hazards with selected demographic variables among computer operators	Self-instructional module was effective in increasing the knowledge and awareness of the computer operators regarding prevention of occupational health hazards
Arumugam, V; Selvam, S; MacDermid, JC 2014	Radial nerve mobilization reduces lateral elbow pain and provides short-term relief in computer users [33]	India (lower middle income)	NRCT (*n* = 41)	Stretches	Physiotherapist directed or guided movements	The purpose of this study was to evaluate the effect of neural mobilisation of the radial nerve on a single occasion in terms of its ability to reduce lateral elbow pain	The mobilisation of the radial nerve resulted in immediate reduction in the pain reported by the participants post-intervention and significant short-term relief in the lateral elbow pain of computer users
Peres, SC Mehta, RK Ritchey, P 2016	Assessing ergonomic risks of software: development of the SEAT [34]	USA (high income)	NRCT (*n* = 166)	– Ergonomic work environment – Workplace structure	Adjustments to the mouse, touch screen and keyboard, taking regular breaks	To develop a self-report ergonomic assessment tool (SEAT) for assessing the risks of software interaction designs and to facilitate mitigation of those risks	Repeated measures analyses of variance showed that participants could discriminate the different strain induced by the input methods and tasks. However, participants’ ability to discriminate between the stressors associated with that strain was mixed. Further validation of the SEAT is necessary but these results indicate that the SEAT may be a viable method of assessing ergonomics risks presented by software design
Levanon, Y; Gefen, A; Lerman, Y; Givon, U; Ratzon, NZ 2012	Reducing musculoskeletal disorders among computer operators: comparison between ergonomics interventions at the workplace [18]	Israel (high income)	RCT (*n* = 66)	– Ergonomic equipment – Ergonomic work environment – Promotive education – Stretches – Breaks and rest periods	– Personal worksite adjustments: adjustment of chair, back rest, lumbar support, handles or arm and table height. The keyboard, screen, lighting were adjusted – Improving work habits: relaxing shoulders, improving sitting habits – Improving muscle activity relaxation – Mini breaks; muscle relaxation for 1–2 s every 5 min – Breaks: stop working for 1–2 min every 30 min and for 5 min every hour, or before the pain usually appears accompanied by a computer announcement – Home programme: stretching and exercises twice a day and taking breaks	This control study aimed to evaluate the efficacy of a workplace intervention for reducing MSDs among computer workers	The intervention programs showed significant reduction of the musculoskeletal disorders’ scores of participants in the intervention groups compared to the control
Singh, A 2019	Shape-Changing Break Reminders for People with Repetitive Strain Injury [64]	Canada (high income)	Qualitative design	Breaks and rest periods	Break reminders	To understand the challenges faced by people with RSI at work, engage with them, and identify their needs from a break reminder system, we reviewed prior work related to repetitive strain injury, interruptions at work, calm technologies, and participatory design	The use of the shape-changing break reminder was found to be an effective solution as now participants were actively trying to incorporate breaks and movement during their workday, further reducing the risks to further development of RSIs
Gerarda, MJ; Armstrong, TJ; Rempel, DA; Woolley, C 2002	Short term and long-term effects of enhanced auditory feedback on typing force, EMG, and comfort while typing [19]	USA (high income)	RCT (*n* = 22)	Ergonomic equipment	Equipment used: A keyboard force monitor. The keyboard force monitor containing three load cells was mounted below a computer keyboard. The plastic cover on the keyboard was removed to decrease the resonance of the keyboard and to remind participants to rest hands or fingers on the keyboards	Part 1: The purpose of this study was to examine and compare the effects of force feedback and EMG feedback on typing force and finger flexor and extensor muscle activity Part 2: The purpose of this study was to examine how long-term auditory feedback would affect typing behaviour, both during and after the presentation of feedback	This research shows that a simple auditory feedback device can reduce typing force and EMG After 1 week of intermittent enhanced auditory feedback there was no difference in typing force or EMG while subjects were typing with or without the enhanced auditory feedback The continued use of auditory feedback did not further reduce the levels of typing force or EMG after 1 or 2 weeks of exposure
Bernaards, CM; Bosmans, JE; Hildebrandt, VH; van Tulder, MW; Heymans, MW 2014	The cost-effectiveness of a lifestyle physical activity intervention in addition to a work style intervention on the recovery from neck and upper limb symptoms in computer workers [20]	Netherlands (high income)	RCT (*n* = 466)	– Preventative ergonomic training – Stretches	Specific equipment not mentioned	To evaluate the cost-effectiveness of a workstyle (WS) intervention and a work style plus physical activity (WSPA) intervention in computer workers with neck and upper limb symptoms compared with usual care	The WS intervention was more effective than usual care in reducing current pain, average pain and worst pain in the past 4 weeks, but the WSPA (work style plus physical activity) intervention was not. This study shows that the WS intervention was not cost-effective for improving recovery but was cost-effective for reducing pain intensity, although this reduction was not clinically significant
Ripat, J; Scatliff, Tom; Giesbrecht, E; Quanbury, A; Friesen, M; Kelso, S 2006	The effect of alternate style keyboards on severity of symptoms and functional status of individuals with work-related upper extremity disorders [21]	Canada (high income)	RCT (*n* = 68)	Ergonomic equipment	Ergonomic keyboard—modified version of the same keyboard designed to reduce activation force, vibration and key travel	– To investigate whether alternate style keyboards were effective in reducing symptom severity and improving functional status for individuals who experience WRUED symptoms – To identify whether symptom severity and functional status improved for users of the intervention keyboards over a 6-month period – To identify whether there was an improvement in clinical measures of WRUED impairment for users of the intervention keyboard groups over a 6-month period – To measure user satisfaction with the intervention keyboards. – To determine whether users of the intervention keyboards were able to maintain typing speed and accuracy with the new keyboards after an initial phase of adjustment	Between-groups analyses indicated that the groups performed similarly on the outcomes of interest. Repeated-measure analysis identified a reduction of symptoms, an improvement in functional status, preference for and increased satisfaction with the intervention keyboards, and maintenance of typing speed and accuracy for both groups
Visschers, VHM; Ruiter, RAC; Kools, M; Meertens, RM 2004	The effects of warnings and an educational brochure on computer working posture: a test of the C-HIP model in the context of RSI-relevant behaviour [14]	Netherlands (high income)	RCT (*n* = 125)	– Preventative ergonomic training – Promotive education	Display warning about participants’ working postures and educational brochure	This study tested whether warnings result in a better working posture with respect to RSI prevention compared with an educational brochure	The computer warning resulted in more position adjustments than the educational brochure and control conditions during the computer task. As people do not often take the time to read a whole brochure, an interrupting warning may be a more optimal way of providing the information needed to perform the desired behaviour
Peper, E; Wilson, VS; Gibney, KH; Huber, K; Harvey, R; Shumay, DM 2003	The integration of electromyography (SEMG) at the workstation: assessment, treatment, and prevention of repetitive strain injury [22]	USA (high income)	RCT (*n* = 27)	– Ergonomic equipment – Preventative ergonomic training – Promotive education – Breaks and rest periods	Ergonomic chairs, document holders, foot supports, visual feedback of the muscle and respiratory patterns, learning to relax neck and shoulders, practicing lower breathing during computer work, incorporating microbreak and larger movement breaks, and ergonomic and work style changes	This paper explores how applied psychophysiology, especially SEMG feedback, offers an approach well-suited to explore discomfort at the computer. After an overview of RSI, the paper focuses upon research studies in the following categories: (1) ergonomic factors that affect musculoskeletal disorders at the workstation, (2) model physiological assessment protocol while working at the computer, (3) the importance of awareness and workstation psychophysiology, (4) psychophysiological prevention/intervention programs for RSI, and (5) summary and implications for enhancing productivity and healthy work styles during computer use	The experimental group showed a significant improvement in subjective rating of symptoms as they breathed significantly slower and diaphragmatically and had reduced upper trapezius SEMG activity. No significant differences were found in deltoid muscle or hand temperature
Ripata, J; Giesbrechta, Ed; Quanburya, A; Kelso, S 2009	Effectiveness of an ergonomic keyboard for typists with work-related upper extremity disorders: a follow-up study [51]	Canada (high income)	Cohort study (*n* = 75)	Ergonomic equipment	Ergonomic keyboard	To investigate whether long-term use of an ergonomic keyboard was effective in reducing symptom severity and improving functional status for individuals who experience symptoms of work-related upper extremity disorders	The results suggest that the use of an ergonomic keyboard may serve to maintain functional status and prevent further development of symptoms over the long term for individuals with mild WRUED. The introduction of an ergonomic keyboard may mitigate the progression of symptom development
Nieuwenhuijsen, E 2003	Health behavior change among office workers: an exploratory study to prevent repetitive strain injuries [52]	USA (high income)	Cohort study (*n* = 40)	– Preventative ergonomic training – Promotive education	– Posters promoting proper postures and preventative activities – Mini-workshops (20 min) and activities of a wellness ergonomic team to promote health and wellness-related activities	To investigate the impact of a multi-component intervention on health behaviour change among office/computer workers in preventing RSI’s	Exposure to a combination of written education materials, hands-on workshops, peer support and reinforcement of safe work postures as well as ergonomic workstations may prevent work-related RSI. Self-efficacy (the belief that one can succeed in health goals), perceived health status, and intention play an important role in that each participant had control over reducing certain risk factors
Holzer, L 2006	Good piano technique: the key to healthy computer keyboarding [53]	USA (high income)	Prevalence study (*n* not reported)	– Ergonomic work environment – Promotive education	Not specified	Modern computer users can learn from pianists about good technique to maintain physical comfort and longevity in their work	Prevent injuries before they start by making a habit of using good posture and ergonomically designed tools at your desk. Consider piano lessons to develop muscles in hands and arms
Munavarah, S; Thenmozhi, R 2016	A study of carpal tunnel syndrome and computer vision syndrome among regular computer users and effect of yogic exercises in them [54]	India (lower middle income)	Cross sectional study (*n* = 100)	– Promotive education – Stretches	Yoga exercises and relaxation techniques	To determine the prevalence of carpal tunnel syndrome (CTS) and computer vision syndrome (CVS) among regular computer users and to study the effectiveness of yogic exercises and relaxation techniques in the improvement of carpal tunnel syndrome and computer vision syndrome among regular computer users	Yogic exercises help to reduce the symptoms of CTS and CVS. Improvement in the reduction was statistically significant in the study and therefore regular computer users with CTS and CVS can be advised to engage in yogic exercises for improvement of computer-related health problems
Palmer, KT; Cooper, C; Walker-Bone, K; Syddall, H; Coggan, D 2001	Effects of forearm and palm supports on the upper extremity during computer mouse use [55]	United Kingdom (high income)	Cross sectional study (*n* = 4889)	Ergonomic equipment	Keyboard	To examine the relationship between upper limb symptoms and keyboard use in a population survey	Regular keyboard use was significantly associated with pain in the shoulders, the wrists and/or hands, but not with elbow pain or sensory symptoms over the same period, or with neck or upper limb pain which had prevented normal activities in the past year
Zuniga, AMF 2015	Muscular, visual and proprioceptive outcomes of computer work with one versus two computer monitors [56]	Canada (high income)	Cross sectional study (*n* = 27)	Ergonomic equipment	Single monitor versus a dual monitor workstation	To quantitatively compare the effects of a standardised 90-min computer task using a single monitor workstation versus a dual monitor workstation on muscular outcomes, as well as on neck/shoulder proprioception/position sense and visual strain in males and females	Results suggest that Dual Monitor work is effective in reducing cervical muscle activity, dissociating cervical connectivity, and maintaining more typical neck/shoulder repositioning patterns, suggesting some health protective effects
Klussmann, A; Gebhardt, H; Liebers, F; Rieger, MA 2008	Musculoskeletal symptoms of the upper extremities and the neck: a cross-sectional study on prevalence and symptom-predicting factors at visual display terminal (VDT) workstations [57]	Germany (high income)	Cross sectional study (*n* = 1065)	– Ergonomic work environment – Breaks and rest periods	Not specified	The aim of the study was to determine the prevalence and the predictors of musculoskeletal symptoms in the upper extremities and neck at visual display terminal workstations	Neck and shoulder symptoms occurred significantly more often than symptoms in the distal parts of the upper extremities e.g., hand, wrist, elbow, forearm when associated with a large amount of typing per day. Thus, the study suggests that preventive measures at VDT workstations should be focused more on neck and shoulder symptoms (e.g., ergonomic measures, breaks to avoid sitting over long periods)
Pascarelli, E 2004	Complete guide to repetitive strain injury [58]	Canada (high income)	Case report	– Ergonomic equipment – Ergonomic work environment Preventative ergonomic training – Stretches	Adaptive equipment, fitting to the equipment, postural training, stretching, mouse placement	– This book is for the reader who wants to benefit from clinical experience with RSI and years of treating people who have it – It was written for people who want to learn how to effectively deal with this illness and are willing to do the work required to get better – How to diagnose and manage repetitive strain injury (RSI)	– Fitting equipment is important: – It is essential to place the body in correct balance to work Improving ergonomics can begin to reverse the discomfort and pain of RSI – Stretching the soft tissues prepares them for mobilisation and strengthening. Stretching can improve muscle balance and diminish the pressure on nerves, joints, and other structures. Stretching should be done regularly and become an integral part of treatment or prevention programs Although mouse placement is critical to prevent arm and shoulder problems, it is the gripping of the mouse that leads to disabling thumb tendinitis – Both medication and psychological counselling can be useful in controlling pain and healing tissue
Smith-Stoner, M 2001	Health tips for computer users [59]	USA (high income)	Case report	– Ergonomic equipment Ergonomic equipment – Ergonomic work environment – Preventative ergonomic training – Stretches – Promotive education	Using a trackball instead of a mouse, split keyboard, wrist support pad Adjusting the equipment for optimal use, stretches, mouse placement or adaptive equipment, psychological counselling	This article reviews health and safety practices related to desktop and hand-held computers This book is for the reader who wants to benefit from clinical experience with RSI and years of treating people who have it – It was written for people who want to learn how to effectively deal with this illness and are willing to do the work required to get better – How to diagnose and manage repetitive strain injury (RSI)	The main conclusion for preventing RSIs is to use light touch when typing, focus on maintaining your wrists in a neutral position when typing and consider using a split keyboard or wrist support The specific intervention strategies relating to Carpal Tunnel Syndrome include performing several wrist exercises, such as moving the wrist in a circular motion, before, during and after typing as well as using a wrist support pad – Adjusting equipment for optimal use is essential to ensure that the body is correctly balanced. – First, it is essential to place your body in correct balance to do your work. Just improving ergonomics can begin to reverse the discomfort and pain of RSI – Stretching the soft tissues prepares them for mobilisation and strengthening. Stretching can improve muscle balance and diminish the pressure on nerves, joints, and other structures. Stretching should be done regularly and become an integral part of your treatment or prevention program. Another source of thumb tendinitis is the mouse. Although mouse placement is critical to prevent arm and shoulder problems, it is the gripping of the mouse that leads to a disabling thumb tendinitis. Both medication and psychological counselling can be useful in controlling pain and healing tissue
Johnson, H 2016	A user-centered ergonomic keyboard design to mitigate work-related musculoskeletal disorders [60]	USA (high income)	Case control study	Ergonomic equipment	Trinity keyboard design (3 planes which can be adjusted as necessary)	To design a slanted keyboard which would encourage a neutral posture when typing while avoiding any negative impact on speed and accuracy and to eliminate the bulky appearance of the current alternative ergonomic keyboard products on the market	The tilt angle and palm rest design of the trinity keyboard reduces forearm pronation when compared with a standard keyboard
McDermott, H; Lopez, K; Wales, B 2004	Computer ergonomics programs [61]	USA (high income)	Case control study	– Ergonomic equipment – Ergonomic work environment – Preventative ergonomic training – Stretches	Take mini breaks, stretching exercises, minimum keystroke pressure, document holder	Under risk-based ergonomics programs, an employer can target interventions toward workers who are in higher risk categories rather than toward the entire employee population to implement preventative ergonomic programmes for preventing RSI	These interventions fostered a culture where employees report discomfort immediately and prevent development of RSI, respond rapidly with appropriate healthcare staff when discomfort was reported to prevent development of RSI And there was reinforcement of proper behaviours through behavioural safety techniques
Cole, DC; Wells, RP & The Worksite Upper Extremity Research Group 2010	Interventions for musculoskeletal disorders in computer-intense office work: a framework for evaluation [62]	Canada (high income)	Qualitative research	– Ergonomic equipment – Ergonomic work environment	Adjustable desks and chairs which met a basic ergonomic standard acceptable to health and safety experts, were purchased to ensure anthropometric fit. Better alignment of mouse and keyboard heights to improve forearm support	We describe a framework for evaluating field interventions – Drawing on work currently in progress, we demonstrate its application in a field intervention to reduce WMSD among office workers at a large newspaper	In order to improve productivity levels and reduce the incidence of RSIs, multi-skilling of team members and ongoing improvement or ergonomic layout of workstation needs to be improved
Meals, C; Koenigsberg, ES 2015	Ergonomic strategies for computer users with upper limb problems [63]	USA (high income)	Qualitative research	– Ergonomic equipment – Ergonomic work environment – Promotive education	Ergonomic optimisation of chairs, keyboards, monitors to allow for neutral positioning of all major joints. Neutralising braces, desktop bumps or pads, specially designed mice and pads. Using 2 different keyboards/mice and alternating	Although ergonomic interventions benefit users, the science supporting them may be poorly understood by both patients and doctors and does not always play a role in the rehabilitation process	Carefully chosen devices may benefit computer users with upper limb problems such as carpal tunnel syndrome but there is little high-quality data to guide decision making in this regard. Ergonomic optimisation relies not only on equipment, but also on education and behaviour modification

^a^According to World Bank Rankings [65]

With consideration of the time frame 2000–2021, the majority of the studies were published in USA, and this amounted to 33% (*n* = 18) of all studies included in this review;Canada and Netherlands yielded 9 studies each.

An overview of types of documents included in the review is that 69% (*n* = 40) were journal articles, 15% (*n* = 9) were research reports, theses constituted 14% (*n* = 8), and the final 2% (*n* = 1) were chapters from a single textbook.

Twenty-four studies specified the daily number of hours spent working on a computer [9, 10, 13, 15–23, 28, 30, 32, 33, 46, 52, 54–57, 64]. A minimum of 4 h per workday was the category reported most frequently by the above-mentioned studies.

**Fig. 2:**
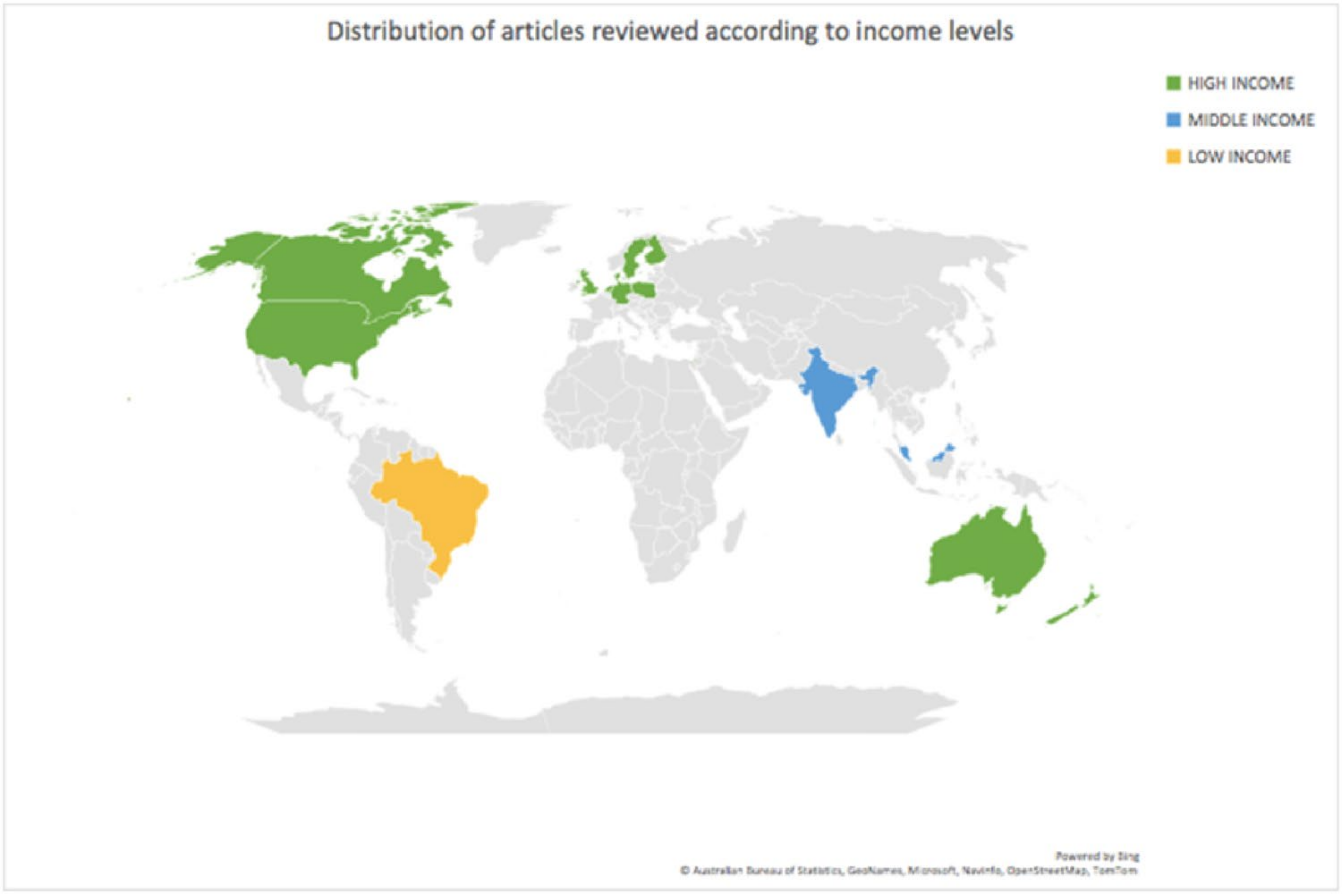
Distribution of studies by income status according to world map

The world map in Fig. 2 represents the distribution of the studies according to country income levels. For the current 2022 financial year, and with reference to the World Bank Atlas Method and GNI per capita index, a country classified as a low-income country has a GNI index of R16 470,87 or less per annum; middle-income country is classified as such an economy, if its GNI index is between R16 470,87 and R200 109,27 per annum [65]. A high middle-income country is classified as such an economy, if the GNI per capita index is R200 109,27 or above per annum [65]. The conversion from US Dollars to Rands was made on the 16th of June 2022 when the exchange rate was $1 = R15,82 [65].

This concludes to 48 studies published in the high-income countries [3, 4, 9–13, 15, 16, 18–27, 30, 34–37, 40–46, 48–64], 8 published from middle-income countries [17, 28, 32, 33, 38, 39, 47, 54], and 2 published from the low-income countries [29, 31].

**Fig. 3:**
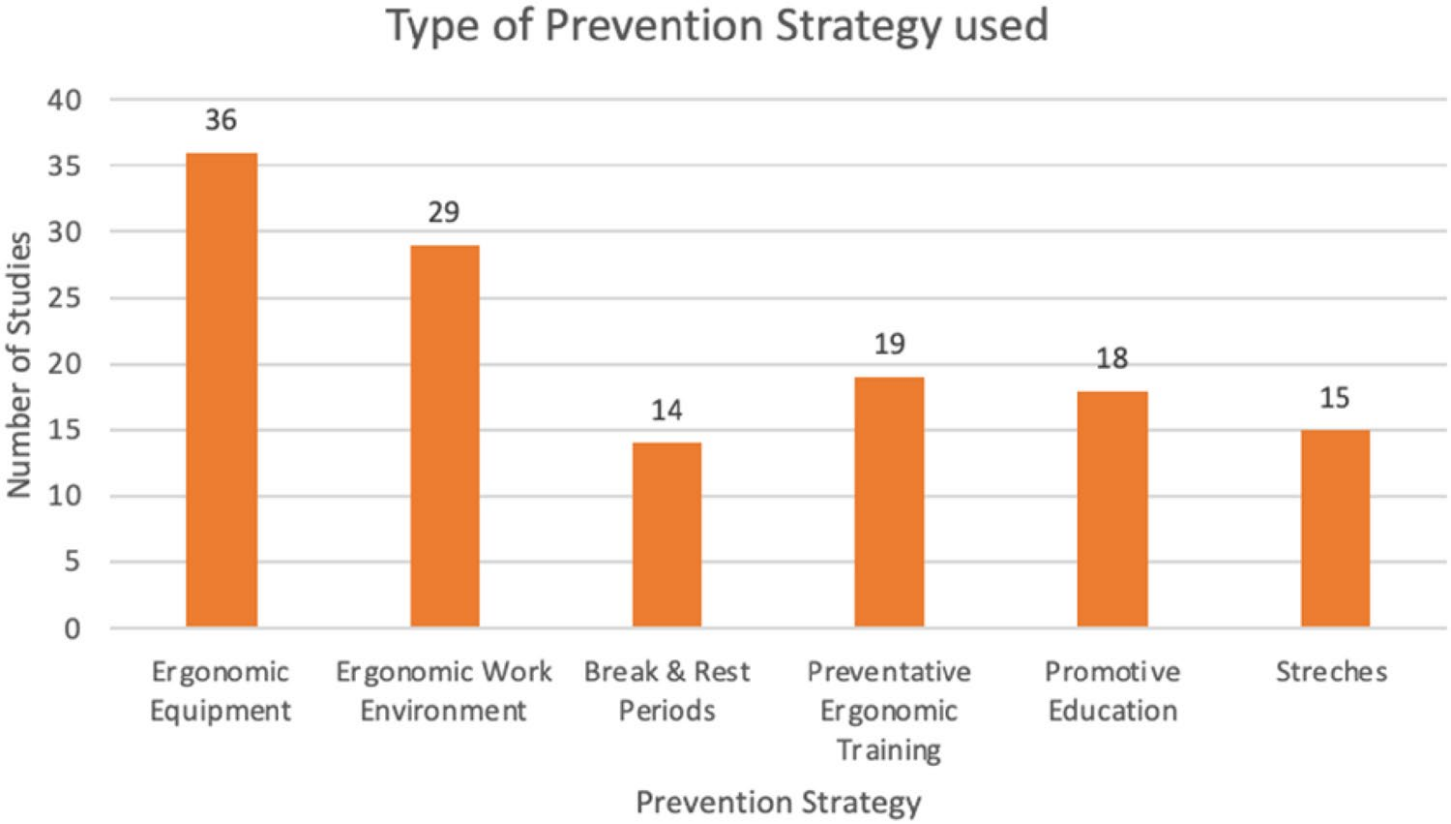
Type of prevention strategy used

Interventions were classified into ergonomic equipment, ergonomic work environment, preventative ergonomic training, promotive education, stretches, breaks and rest periods, as can be seen in Fig. 3. This was an exhaustive list as all documents could be classified into one of these categories.

The most common intervention strategy found to prevent RSI in the UL in computer users was ergonomic equipment (*n* = 36) [3, 9, 10, 16, 18, 19, 21, 24, 26, 27, 30–32, 35–45, 48–51, 55, 56, 58–63]; and the least common therapeutic intervention was breaks and rest periods (*n* = 14) [4, 11, 18, 22, 23, 25, 29, 30, 38, 39, 43, 49, 50, 64]. The ergonomic equipment can be further classified into using an ergonomic mouse or keyboard and using forearm support.

The second most common prevention strategy (*n* = 29) [12, 15, 18, 19, 21, 22, 25, 28, 30–32, 34, 38, 39, 42, 46, 48–51, 53, 56–63], with reference to Fig. 3, was ergonomic work environment which included personal worksite adjustments and adaptive furniture such as the adjustment made to the height of the office desk and chair, dividing the workload and desk organisers.

Preventative ergonomic training as a therapeutic intervention strategy were reported in 19 studies [3, 12, 14, 20, 22, 23, 28, 30, 36–39, 42, 43, 46, 49, 50, 52, 58]—this includes exercises, policy, behavioural therapy, and preventative activity workshops. Eighteen studies reported promotive education as an intervention to prevent RSIs in computer users [4, 9, 13–15, 17, 18, 22, 28–30, 32, 36, 39, 50, 52, 59, 63]—this included posture training, health and wellness training, education and teaching relaxation techniques.

## Discussion

The discussion section is structured under the research questions developed during the design of the scoping review, towards demonstrating how they were addressed.

*What are the therapeutic approaches used to prevent RSIs in the UL in computer users within the workplace environment in the twenty-first century*?

The main therapeutic approaches for the prevention of RSI’s of the UL in computer users extracted from the 58 studies in the review are:

1. Ergonomic Interventions

Current research suggests that participative ergonomics and training are essential for the successful training of employees as well as individualised evaluations of computer workstations [40]. Use of a wrist support pad for the prevention of Carpal Tunnel Syndrome is recommended [59]. The use of a corner workstation offering forearm support increased the variability of muscle activation patterns [25].

2. Mouse Design

A mouse which requires a neutral forearm posture and reduces pronation may have a protective effect on the ulnar nerve at the level of the wrist [10]. A mouse which places the forearm in a pronated position could increase the risk of musculoskeletal symptoms whereas, one which encourages a neutral position could allow for a more relaxed posture of the forearm thereby resulting in a reduction in RSI [53].

3. Rest Breaks

Favourable effects on discomfort or complaints have been found for rest breaks of 5 and 10 min after every hour of work, as well as incorporating 30 s microbreaks at 20-min intervals [23]. Klussmann, Gebhardt, Rieger report that it is more beneficial to take microbreaks according to a fixed schedule of every 20 min for the prevention of back, shoulder and forearm conditions [57].

When disseminating results of the different types of therapeutic intervention strategies according to the economic status it was discovered that ergonomic equipment was the most common type of intervention in the high-income countries. In middle income countries, stretches were seen as the most common type of therapeutic intervention strategy used to prevent the development of RSIs amongst computer workers. In low-income countries there was an even distribution among all therapeutic interventions aimed at preventing RSIs.

4. Keyboard Modifications

Alternative keyboard studies were introduced for the reduction of pain, fatigue or other clinical effects relative to standard keyboards [39].

5. Positioning

The following approaches to positioning hands on the keyboard with reference to ergonomics have been recommended: (1) Use light touch when typing; (2) focus on maintaining your wrists in a neutral position when typing; and (3) consider using a split keyboard or wrist support. Exercise breaks such as performing several wrist exercises like moving the wrist in a circular direction at regular intervals is recommended for the prevention of Carpal tunnel syndrome [59].

*What factors contribute towards the implementation of therapeutic approaches aimed at preventing RSIs of ULs amongst computer users within the workplace*?

Proactive management and implementation of company policy is required for successful implementation of prevention strategies. Ongoing improvement and monitoring of ergonomic layout of workstations and multi-skilling of team members is required to reduce the incidence of RSIs which relies on company policy for implementation and allocation of budget [15]. Policy makers and the media play a pivotal role in educating the population about the importance of ergonomics in the prevention of RSI’s [58].

*What are the trends and gaps in literature of in twenty-first century relating to therapeutic approaches to aid prevention of RSI of the UL in computer users*?

Future research should investigate the determinants of pain reduction and recovery from upper limb symptoms so that (cost-)effective interventions can be developed. There is a need for further studies to establish the association between computer mouse use and RSI, and to investigate the influence of computer mouse design on posture and muscle activity. More research is needed to address psychological stressors in an individual person’s life and overall workplace environment. Further research should investigate the effectiveness of ergonomic keyboards in preventing the evolution of RSIs in users that do not experience symptoms and how the design of a mouse could contribute to the evolution of RSIs in conjunction with the ergonomic keyboard.

Moreover, future research should focus on providing high quality studies detailing the effects of office ergonomic interventions on musculoskeletal or visual health. Further study to establish whether it is necessary to assign a maximum typing duration to reduce the risk of RSIs. Future research should aim to provide evidence on how to systematically minimize the effect of risk factors and other workplace barrier. Future studies should include middle and low-income countries, in order to have more diverse and inclusive prevention strategies that are effective as this is not a true reflection of the current state of practice in middle- and low-income countries. Future studies need to fit intervention into participants’ existing work roosters/schedules. Studies should include interventions with a holistic approach that considers individual, environment and occupation factors involved. There is a need for high quality studies around this topic as only a few are currently peer-reviewed. Research should focus on planning and recommending further and frequent implementation of interventions in the workplaces.

## Strengths and Limitations

The study results were strengthened by reviewing each study independently by two separate members of the research team. Consequently, a single study was considered against eligibility criteria at least twice, whereby bias was reduced. Utilising well known and trusted databases and MeSH terms during searches was advantageous for expanding on included documents while maintaining the relevancy. The 22-year timeframe considered during the review allowed for the observation of the rapid advancement of technology and how this affects this field of study.

The broad timeframe can also be viewed as a limitation as the wide timeframe, may conversely highlight approached that may be ‘out of date’ considering the rapid advancement of technology in the twenty-first century. A further limitation is the lack of inclusion of studies for which full text could not be obtained, despite best efforts by the research team. Moreover, there were scoping and systematic reviews included in the current review which can be viewed as a limitation. We however considered the overarching results of the included reviews in an attempt to address the research objectives.

## Conclusions

The scoping review has explored the evidence across a range of study designs, to systematically map the literature available on the therapeutic approaches for the prevention of UL RSIs in computer users within the workplace. Using the Arskey and O’Malley framework to ensure that analysis was rigorous, valid, and reliable, allowed the identification relevant key concepts and gaps in the available research.

The focus of the study has been on the state of the research activity as opposed to the quality of the research. Ergonomic equipment was the most prevalent intervention strategy to prevent UL RSIs amongst computer users and breaks and rest periods were deemed to be the least used intervention. The specific strategy that was mentioned by the greatest number of studies was personal worksite adjustments (*n* = 26) and the second is the use of an adapted or adjusted keyboard (*n* = 20).

A multi-component approach including policy, ergonomic adjustments, behaviour modification, promotive training and correct posture emerged as viable approaches.

In conclusion, the review provides a basis for therapists, researchers, and company stakeholders to establish or expand existing therapeutic approaches for the prevention of RSI of the UL in computer users.

## Declarations

### Guarantor

AdW.
